# 

**Published:** 1884-07

**Authors:** 


					﻿vZik
JULY, 1884.
NO. 7.
THE
HOMEOPATHIC pHY^GlAJi,
A MONTHLY JOURNAL OF MEDICAL SCIENCE.
“ Seek the Truth: come whence it may, cost what it wiU."
E3. CT. LEE, JVE. ZD.,
EDITOR.
CONTENTS:
(See inside of Cover.)
PHILADELPHIA:
No. 2109 CHESTNUT STREET.
nSTU’W YORK;
A. L. CHATTERTON, PUBLISHING COMPANY, Publisher’s Agents.
LONDON:
ALFRED HEATH & CO., Sole Agents for Great Britain,
114 Ebury Street, Eaton Square.
Yearly Subscription, $2.50, invariably in advance,
Entered at the Philadelphia Post-Office as Second-Class Mail Matter.
				

